# A Fuzzy Model to Interpret Data of Drive Performances from Patients with Sleep Deprivation

**DOI:** 10.1155/2012/868410

**Published:** 2012-08-30

**Authors:** Pasquale Sena, Paolo Attianese, Francesca Carbone, Arcangelo Pellegrino, Aldo Pinto, Francesco Villecco

**Affiliations:** ^1^Department of Pharmaceutical and Biomedical Sciences, University of Salerno, Via Ponte Don Melillo, 84084 Fisciano, Italy; ^2^Department of Industrial Engineering, University of Salerno, Via Ponte Don Melillo, 84084 Fisciano, Italy

## Abstract

The search for safe vehicles is increasing with both diffusion of high traffic density over the world and availability of new technologies providing sophisticated tools previously impossible to realize. Design and development of the necessary devices may be based on simulation tests that reduce cost allowing trials in many directions. A proper choice of the arrangement of the drive simulators, as much as of the parameters to be monitored, is of basic importance as they can address the design of devices somehow responsible for the drivers safety or, even their lives. This system setup, consisting of a free car simulator equipped with a monitoring system, collects in a nonintrusive way data of the car lateral position within the road lane and of its first derivative. Based on these measured parameters, the system is able to detect symptoms of drowsiness and sleepiness. The analysis is realized by a fuzzy inferential process that provides an immediate warning signal as soon as drowsiness is detected with a high level of certainty. Enhancement of reliability and minimisation of the false alarm rate are obtained by operating continuous comparison between learned driver typical modalities of operation on the control command of the vehicle the pattern recorded.

## 1. Introduction

Development of new systems aimed at increasing the equipments for active safety of motor vehicles is draining more and more attention in both academies and industries, involving scientists from many fields ranging from engineering to medicine. Actually, attention is focused on systems or devices able to account for all possible risks either deriving from an unfavourable external environment or from improper drivers psychophysical conditions. These latter are the real challenge to win, as the current approach possible is to intervene as soon as there is evidence of any driver difficulty that, however, often comes out when safety is already compromised.

Microsleeps are among the most critical occurrences. Data from the National Statistics Institute (ISTAT) on this subject (as of 20.11.2008 for the year 2007) say that accidents due to physical or mental conditions of the driver correspond to approximately 3.8% of the total. Among these, the fraction of accidents officially related to classic microsleeps is 0.26% [1]. However, the analysis is misleading as it underestimates the real effect of sleepiness. 

It is a matter of fact that the real nature of traffic accidents is multifactorial. This hinders a correct evaluation of the contribution of sleepiness as much as of microsleeps. When considering the role of sleepiness, this is often masked from other more evident factors such as excessive speed, or weather conditions or state of the vehicle, becoming thus a cofactor whereas somnolence should be considered the final common pathway of the other factors listed above.

To date, this interaction is not deeply studied although recent studies outline, with increasing relevance, characteristics that can be considered typical of situations with excessive sleepiness, and point out that drowsiness may be, in fact, an important risk factor in the occurrence of accidents or may contribute, even to a less extent, to the increase of the mortality index in car accidents [2–5]. It has to be noted that the percentage of traffic accidents attributed to sleepiness considerably vary from country to country, due to differences in instruments and methods of data collection used by the local authorities. Other factors of variability may lead to the underestimation of the phenomenon, such as type of population studied, different definitions of sleepiness, objective difficulty to assess the degree of sleepiness of the driver at the accident site. Actually, most of the available data come from inferential evidence.

Accidents caused by microsleeps have some peculiarities, that may help in a classification [6]. In fact, these accidents mainly occur at night time, early morning, or middle afternoon and, more frequently, during monotonous driving conditions such as in highways or when the driver is alone in the vehicle. All these accidents show similar dynamics, with a single vehicle involved leaving the road regardless of weather or traffic conditions, without any trial of the driver to avoid the accident. 

Thus, microsleeps just are the emerging tip of a progressive phenomenon characterized by a gradual reduction in the level of supervision, which in turn induces a continuous decrease in psychomotor performances and, consequently a reduction of reaction times, a diminished risk perception and a decrease of attention which may have a value in determining the occurrence of a traffic accident. This specially occurs when driving is monotonous and repetitive, favouring the emergence of a latent sleepiness which is often not perceived by the driver.

This partial drowsiness, can be also induced or facilitated by a variety of abnormal conditions, dysfunctions and diseases, either common or rather widespread in the general population. Pregnancy, jetlag, excessive exercise, or intake of abuse of psychoactive drugs such as alcohol and marijuana, can affect alertness causing excessive sleepiness, impaired reaction times, decreased ability to recognize environmental signals, or impaired judgment. The same happens with sleep disorders, such as insomnia, restless legs syndrome, or the sleep apnea. This deterioration of driving skills can also be promoted by specific pathological conditions such as hypothyroidism, migraine, depression, and certain infectious diseases, such as the common flu.

In addition to drugs that act specifically on the brain (sedatives, hypnotics, antidepressants, and neuroleptics), some antihistamines, including those of more recent introduction, can have side effects like sedation but different individual responses. The drowsiness has also been reported with drugs widely used as the nonsteroidal anti-inflammatory, for example, diclofenac or indomethacin, and antihypertensives, especially beta blockers.

All these conditions leading to a decrease of attention and concentration during driving and reduced ability in evaluating the risks (e.g., from excessive speed) confirm the multifactorial nature as much as the complexity of the system driver-vehicle environment and outlines the benefits driving safety that could derive from an approach addressed to evaluate the physiopathological conditions of the driver rather then to wait for his failure.

## 2. The Need for Prevention

As above noted, the strategy of many automobile industries for active prevention of accidents from sleepiness is to install inside the vehicle purposely designed devices able to verify the state of vigilance of the driver and to warn him in case of risks.

People driving with excessive sleepiness make great efforts to stay awaken so that the phases of the sleep onset at drive are not comparable to those at home. In fact, the physiological events preceding the sleep show different durations and sequences in the two cases, making data currently collected hard to interpretate.

There is evidence that others parameters could be considered, as frequency and amplitude of the sudden turns of the steering wheel, as they appear able to offer a good indication of the level of sleepiness, although changes in the vehicle direction can sometimes depend on ability and experience of the driver. Measure of frequency of blinking seems also to be promising in terms of effectiveness and reliability.

A risk holds when using the parameters above to define a safety threshold: the related devices could become themselves a source of further risk, as a driver who feels like falling asleep might decide to keep on driving, relying on the safety devices intervention.

Furthermore, most of the approaches currently used in the development and design of driver monitoring devices for detection of conditions of drowsiness are based on the identification of the “dozing” or of a condition of marked drowsiness so that intervention are possible only at the end of the progressive decay phase which, in fact, precedes the onset of sleep.

This paper aims at pointing out the possibility of developing system based on the idea that the progressive decaying of drivers ability to appropriately react to external stimuli rather than its final effect should be monitored, making thus more effective any safety device that in this way could evidence the risk situation largely in time.

## 3. Analysis of the Multifactorial System

Driving a vehicle, is a very complex task that requires a high degree of the neuromotor coordination and the development of a number of appropriate, well-balanced abilities.

The driving ability is primarily attributable to a strong propensity of the driver to develop proper automate mechanisms of control of the vehicle and to establish a continuous monitoring through a number of proprioceptive feedbacks. Driving is thus a complex multifactorial and multiparametric phenomenon that obeys to the principles of neurophysiology, which state that the central nervous system (CNS) does not deal with single muscles, but with a general coordinated movement. Thus, the simplest motor response (e.g., the activation of arts to control the vehicle) actually corresponds to a complex motor act. Moreover, activation and space time organization of the motor response to control steering wheel, pedals, and gear shift depend on the continuous flux of exteroceptive information: visual, auditory, haptic, and vestibular. Driving can thus be defined a built-in complex motor acts, that activates integrate systems and central and affective-emotional components, through peripheral proprioceptive afferents. Any alteration of this complex system due to an inefficiency in a few simple districts, will affect the final result, that is, the safe control of the vehicle.

Following the considerations above, any system capable of nonintrusively monitoring the performance of the driver will need a detailed model of the vehicle-driver couple. 

In particular, the vehicle knowledge has to be associated to the knowledge of the environment the vehicle operates in, being the latter the most difficult part. It is enough to consider, for example, the uncertainty introduced by the presence of other vehicles that move and can suddenly change direction to outline the difficulty of finding probabilistic mathematical functions allowing an easy formulation of the model. These difficulties are also caused by the vehicle interaction with the environment, as is the case of the vehicle dynamics changing for a road surface wet, or of the measurements that may vary with light and geometric or physical properties of the objects.

Therefore, the knowledge basis to define the model must necessarily rely on a simultaneous analysis of all the different parameters, involving both the driver-and the vehicle-related. 

However there is a lack of standardization of the measured variables that makes hard even to decide the parameter to be monitored.

This system should be based on a series of continuous measurements of variables serving as indicators of the driver performance (e.g., steering wheel movements), and on their processing to decide the permission to drive.

## 4. The Experimental Setup

A free car simulator for noncommercial use named Racer (Dolphinity) running on a gaming PC was used to collect data of driving performances. The simulator offers to driver a scenery with a ground level first person perspective view of a highway with no other cars.

The “quick race” mode of the simulator was used, permitting logging of all data of interest. Subjects moved through the environment in a FIAT Punto GT, controlled using a compatible game controller (Thrustmaster t500 rs) consisted of a steering wheel of 30 cm of diameter, 3 pedals equipped with digital encoders, and a gear shift with H pattern.

A modification was employed which ensured that data regarding vehicle parameters and action of the driver on the control commands were continuously acquired and stored. 

A separate PC, linked to the PC running the physical engine of the simulator, read in real time the data logged and processed the data to work out the final score.

## 5. Development of an Experimental Monitoring Device

The idea is to develop an on-board detection system capable of monitoring the driver and formulate an algorithm able to analyze data and decide when the driving ability is materially impaired by drowsiness.

The ideal monitoring system should integrate specific measures of the drive performance with direct possibly noninvasive measurements of the psycho-physiological state of the driver together with measures of reaction time of secondary tasks could be added to evaluate the status of the driver decide consent or denial to drive.

In fact, to an early detection of the condition of drowsiness, the variables must be analyzed in real time and in a combined way because no individual measure appears to be sufficient for a reliable detection of somnolence.

The choice of variables in this work has been made on the basing on particular considerations. The monitoring variable to evaluate the driven condition must necessarily be easy to measure, of practical application, and of an adequate sensitivity. They must also be repeatable and must be able to describe the process on the basis of objective observable parameters. Therefore, a system has been developed that during a simulated driving session records, by a logging device data of the relative position of the car center line and of the lane edge, and compares the driver decisions to the ideal trajectories and options by a controller. Parameters such as the movement of the pedals and of the steering wheel are simultaneously measured and associated to data recorded. 

## 6. The Algorithm

The approach used to develop an algorithms with a high detection accuracy has been to operate mathematical optimization techniques using multiple regressions and linear discriminant analyses. One consider that, although the algorithm is formulated to provide the best performance when all the all the input signals are validated, one or more of the input signals could be missing (e.g., a reliable estimate of the road limits). Therefore, the system has been set to discriminate the valid signals and to ensure that the detection algorithm can always be able to operate.

The algorithms optimization for the detection of somnolence requires a measure of the limit threshold, or in any case of a specific marker that can establish the definition of drowsiness. This may be based on physiological variables, on measures of performance, or on subjective parameters, that have to be sufficiently repeatable in a given set of experiments so that the detection algorithm can be “trained” to determine the value of the alarm threshold definition. In particular, this latter must be adjustable to allow a turning of the system sensitivity according to the different driving conditions.

Note that a baselining is necessary for the algorithm to obtain measures for the individual driver comparable with all data collected.

Furthermore, the algorithm, to be truly effective in identifying the condition of drowsiness, should indicate when the somnolence level has exceeded a prespecified threshold, and this cannot be done through the evaluation of a single parameter, but by a linear combination of the different available measures.

## 7. The Analysis System

The definition of the input parameters used to measure the driving performance is independent of the identification of variables that were actually related to sleepiness. In the literature there are several studies that report the changes in driving performance due to sleep deprivation through various levels of propensity to sleep [7, 8].

Results show a continuing decline of the driver performance with the progression of the state of drowsiness and the driver quality continuously reduces.

In all of these studies, the lateral control is used as a key parameter for the assessment of the driving quality. In particular, the ability to control the horizontal movement of the vehicle and to keep it properly and safely within the lane, has proved to be an excellent indicator of the driving performance.

**Figure 1:**
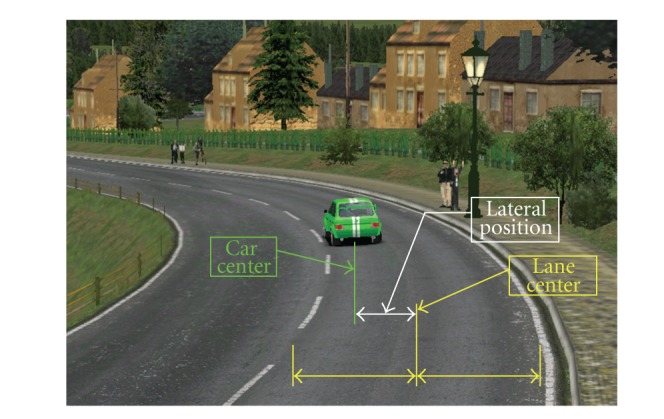
Means of lateral position.

The effectiveness of the lateral control operated by the driver can be expressed by calculating the standard deviation of the lateral position (Figure 1), defined as the horizontal position of the vehicle within the lane, determined with respect to a specific point of the vehicle and the road.

In most cases, the lateral position is determined by calculating the distance between the central axis of the vehicle and the center of the lane. When these two axes are aligned, the vehicle is located in an ideal position. When the vehicle is moving within the lane, either left or right, the deviation from the lane center, is measured by the so-called deviation of lateral position.

In addition to the lateral position (LP) is acquired and processed also the first derivative (LP′) that describes the changes in lateral velocity and is defined such that positive values correspond to lateral movement of the car toward the left side of the lane limit, while negative values correspond to movements toward the right limit.

The magnitude of the drift to one side and its relationship with the actions made on the steering wheel to compensate for, can provide useful information on both the driving quality and style, defined as the tendency of drivers to over- or under-estimate the lateral drift and the greater or lesser propensity to operate correctly on the steering wheel.

### 7.1. The Fuzzification of the Inputs

Both lateral position and the drift are expressed as positive values if the vehicle has moved or drifted to right edge and negative if to the left edge of the roadway. Both variables are fuzzyfied using 5 fuzzi sets that describe the range values of −2 to +2 meters for the lateral position and −20 and +20 centimeters for the drift as follows:

- Sx +: very left;
- Sx: left;
- C: central;
- Dx: a right;
- Dx +: very right.

**Figure 2:**
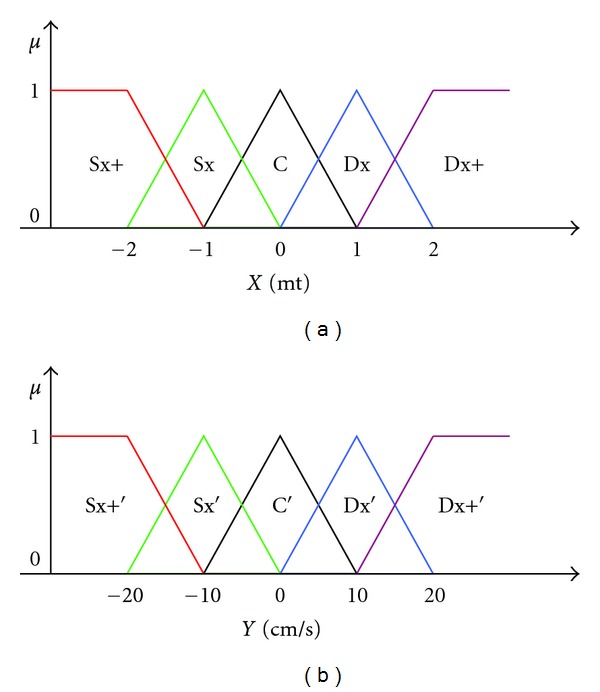
Membership functions of inputs.

The fuzzy sets are reported in Figure 2.

### 7.2. The Inferential Matrix

Using an inferential matrix, that includes as inputs lateral position and its first derivative, is generated a judgment (**J**) on the quality of the vehicle lateral control operated by the driver, in the specific time interval delimited by the time step of signals acquisition.

This matrix considers “safe” the associations of inputs that bring the vehicle to converge towards the center of the lane, whereas are evaluated “unsafe” those that lead the vehicle to diverge from the center of the lane and, then, can lead to go off track.

The inference appear as follows:


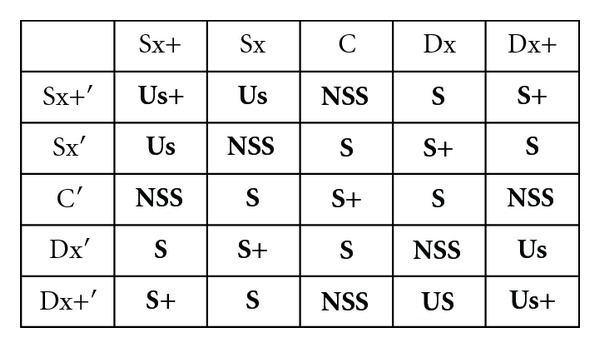


If **LP** is *X* And **LP** ^**′**^ is *Y* Then **J** is *Z*

The possible outputs of the system are 5:

- Us+: very unsafe;
- Us: unsafe;
- NSS: not so safe;
- S: safe;
- S+: very safe.

### 7.3. The Defuzzification

According to the inferential rules activated in the matrix, a crisp value is obtained corresponding to the final score attributed to the driver at that given time of the driving.

This value is obtained by applying the method of the center of the areas to the fuzzy sets activated, according to membership functions similar to those used for the fuzzification of the inputs and will assume value in the range 0–100.

**Figure 3:**
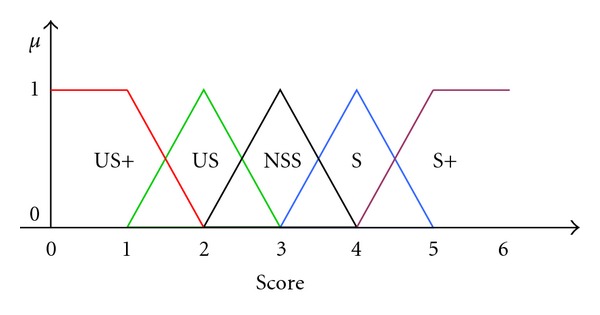
Membership functions of outputs.

The fuzzy sets for the judgment output are reported in Figure 3.

## 8. The Evaluation of the Driving Style

Crisp values of judgments generated are progressively stored in a dynamic database. An external computational unit, linked to the database, calculates in real time the normalized statistics distribution of the judgment value, that is used as a reference to evaluate the performance of the driver in normal condition.

## 9. The Detection of the Driver Impairment

This unit performs a statistical comparison between the distribution stored in the database containing the reference for normal condition and the distribution recorded in the last 10 minutes of driving to detect any significant difference. 

The system also works out the trend of variation of the judgment value, pointing out the trends describing a progressive deterioration of driving quality, expressed as a continuous reduction of the score of judgment.

## 10. Conclusion

The system that we developed here provides a valuable tool for exploring the possibility of implementing an on-board device to detect driver drowsiness and improve driving safety. 

Our analysis algorithm allowed the system to carefully control the typical patterns of actions made by a specific driver in normal conditions and to detect significant differences in those patterns due to incoming of drowsiness conditions. 

The system could benefits of advantages associated with the use of a specific model of steering control whose design is closely guided by human data, instead of a static threshold to quantify the limits of driver safety performance under sleepiness conditions.

The system seems to be very promising for further use as an effective safety device and appears also able to allow understanding of the driver behavior and the relationship holding between the driver's sensory cues and his consequent actions. 

The use of baseline recording of “natural data points” reduces the influence on data analyses of variability in driver.
